# The role of researchers in disseminating evidence to public health practice settings: a cross-sectional study

**DOI:** 10.1186/s12961-016-0113-4

**Published:** 2016-06-10

**Authors:** Allese B. McVay, Katherine A. Stamatakis, Julie A. Jacobs, Rachel G. Tabak, Ross C. Brownson

**Affiliations:** College for Public Health and Social Justice, Saint Louis University, 3545 Lafayette Avenue, St. Louis, MO United States of America; College of Public Health, University of Kentucky, Lexington, KY United States of America; Prevention Research Center in St. Louis, Brown School, Washington University, St. Louis, MO United States of America; Division of Public Health Sciences and Alvin J. Siteman Cancer Center, School of Medicine, Washington University, St. Louis, MO United States of America

**Keywords:** Dissemination, Public health, Practice settings, Researchers, Barriers

## Abstract

**Background:**

Evidence-based public health interventions, which research has demonstrated offer the most promise for improving the population’s health, are not always utilized in practice settings. The extent to which dissemination from researchers to public health practice settings occurs is not widely understood. This study examines the extent to which public health researchers in the United States are disseminating their research findings to local and state public health departments.

**Methods:**

In a 2012, nationwide study, an online questionnaire was administered to 266 researchers from the National Institutes of Health, the Centers for Disease Control and Prevention, and universities to determine dissemination practices. Logistic regression analyses were used to examine the association between dissemination to state and/or local health departments and respondent characteristics, facilitators, and barriers to dissemination.

**Results:**

Slightly over half of the respondents (58%) disseminated their findings to local and/or state health departments. After adjusting for other respondent characteristics, respondents were more likely to disseminate their findings to health departments if they worked for a university Prevention Research Center or the Centers for Disease Control and Prevention, or received their degree more than 20 years ago. Those who had ever worked in a practice or policy setting, those who thought dissemination was important to their own research and/or to the work of their unit/department, and those who had expectations set by their employers and/or funding agencies were more likely to disseminate after adjusting for work place, graduate degree and/or fellowship in public health, and the year the highest academic degree was received.

**Conclusions:**

There is still room for improvement in strengthening dissemination ties between researchers and public health practice settings, and decreasing the barriers researchers face during the dissemination process. Researchers could better utilize national programs or workshops, knowledge brokers, or opportunities provided through academic institutions to become more proficient in dissemination practices.

## Background

Despite the numerous advances made in clinical and public health research over the past few decades, discontinuity still exists in the process of disseminating and implementing research discoveries into practice settings [1–3]. The dissemination and implementation of these discoveries are important in working towards improving the population’s health [4, 5]. However, the most effective interventions are not always utilized in practice settings. Depending on the setting, these interventions may not always be feasible, but the presence of underutilization still raises concerns and highlights the importance for researchers to design studies that include dissemination elements [1, 6–12]. Dissemination of research findings is frequently tailored to specific settings or subsets of the population, which further stresses the need to design for such a process during the conceptual phase of a study in order to successfully reach the targeted destination [7, 13].

Closing the gap between research and practice to allow for successful dissemination has proven difficult [14]. Wilson et al. [15] surveyed applied health services and public health researchers about dissemination practices and found that less than one-fourth of these researchers had a person or team in their department dedicated to dissemination. Furthermore, less than one-fourth of the researchers in the study reported that they had a formal communication or dissemination strategy [15]. Infrastructure changes, such as incorporating mediators, to better communicate and deliver research interventions to public health practice may aid in bridging the gap between research and practice settings [16]. Additionally, some funding agencies (e.g. The National Science Foundation, programs within the National Institutes of Health, and the Robert Wood Johnson Foundation) state that dissemination of research results is a priority and/or expect their grant recipients to disseminate their work to relevant parties [17, 18].

Public health research often focuses on applied studies that offer a high potential for translation of subsequent research findings into practice [1, 19]. Many public health-trained researchers have experience in practice settings either through previous work experience, or as part of public health degree program requirements (e.g. during an internship) [19]. In theory, this practice experience should increase the likelihood of engaging stakeholders, particularly public health department administrators and/or staff, in the research process. Given the value of stakeholder engagement in research and evaluation, there is likely room for improvement when public health researchers are designing studies or preparing findings for dissemination [20–22].

The question of whether public health researchers are involved in dissemination, specifically to practice settings, still remains. There are varying interpretations of dissemination in the literature, but the term dissemination was defined in the current study as, “*an active approach of spreading evidence-based information to the target audience via determined channels using planned strategies*” [6]. The purpose of this study was to examine the extent to which public health researchers are disseminating their research findings to United States state and local public health departments, and the individual- and organizational-level facilitators and barriers that impact whether this dissemination occurs.

## Methods

### Study sample

Sampling for this cross-sectional study began by conducting a PubMed search of lead authors from the top 12 public health journals, sorted by impact factor, within the category “public, environmental and occupational health.” Authors, with a United States affiliation, of publications dated from October 1, 2008, to October 1, 2011, were included. Publications that were considered commentaries, biographies, historical articles, classical articles, reviews, meta-analyses, and webcasts were excluded. Specifying the author’s affiliation, this search yielded 91 CDC researchers and 100 intramural NIH researchers. Of the researchers not affiliated with the CDC or NIH, 200 names were randomly sampled from the PubMed search. In addition, names of principal investigators or doctoral-level project managers were acquired from 37 university Prevention Research Center (PRC) websites, and 100 researchers were randomly sampled. Fifty-seven NIH grantees conducting dissemination and implementation research were procured from program announcements in the RePORTER database. After all inclusion and exclusion criteria were applied, a list of 548 researchers was compiled, and the survey was successfully sent to 488 (exclusions included emails that failed to deliver, duplicate names that were not previously identified, and contacts who were deceased or disabled). Human participant approval was obtained from the Washington University institutional review board. Participation in the survey was characterized as voluntary and researchers could refuse to participate if they wished. A more detailed description of the sampling process was published previously [6].

### Questionnaire

The questionnaire was adapted from a study conducted in the United Kingdom by Wilson et al. [15], which aimed to determine how public health researchers in the United Kingdom were disseminating the results of their research and whether dissemination extended further than academic publication. They conducted a systematic review of conceptual frameworks and based their instrument on the common key elements from this review [15]. For the current study, relevant domains and previous work guided the adaptation of the survey [15, 23–25]. For clarity and ease of interpretation, a definition for the term dissemination was provided in the survey. The 35 question instrument was administered online using survey software developed by Qualtrics (Qualtrics, 2012, Provo, UT) [26]. The survey remained open from January 10, 2012, through March 6, 2012. The total number of completed surveys was 266, leading to an overall response rate of 54.5%, which was only slightly lower than the expected response rate of 60%.

### Measures

The primary outcome of interest was the dissemination of research findings to United States local health departments, United States state health departments, or both (further abbreviated as LSHDs), which respondents indicated in response to the question: “To which of the following non-research audiences have you disseminated the findings of your research?” Other response options besides United States state or local health departments included United States federal agencies, international governmental agencies, international non-governmental organizations, elected officials, non-profit organizations, healthcare providers, health research funders, the target population of the respondent's research, the media, the general public, and other (respondents were given the opportunity to specify another audience in an open text field). Additional covariates of interest included work place (University, NIH, CDC, and other (respondents were given the opportunity to specify their work place in an open text field if other was chosen)), the presence of a graduate degree and/or fellowship in public health, year the highest academic degree was received (categorized as less than 10 years ago, 10–20 years ago, and greater than 20 years ago), and facilitators and barriers to dissemination.

**Table 1:** Respondent characteristics by dissemination to LSHDs, 2012

		Disseminate Findings to LSHDs			
		Yes	No		
	(n = 266)	(n = 154)	(n = 112)		
Respondent characteristics	n (%)	n (%)	n (%)	crude OR (95% CI)	aOR (95% CI)
Work place					
NIH	25 (9)	8 (5)	17 (15)	Reference	Reference
University, not a PRC	109 (41)	45 (29)	64 (57)	1.5 (0.6–3.8)	1.2 (0.5–3.1)
University, PRC	63 (24)	56 (36)	7 (6)	17.0 (5.4–53.7)	10.8 (3.3–35.9)
CDC	34 (13)	29 (19)	5 (4)	12.3 (3.5–43.8)	9.5 (2.6–34.8)
Other/Missing	35 (13)	16 (10)	19 (17)	1.8 (0.6–5.2)	1.4 (0.5–4.3)
Degree in Public Health					
No	151 (57)	77 (50)	74 (66)	Reference	Reference
Yes	115 (43)	77 (50)	38 (34)	2.0 (1.2–3.2)	1.8 (1.0–3.3)
Year degree was received					
Less than 10 years ago	70 (26)	27 (18)	43 (38)	Reference	Reference
10–20 years ago	99 (37)	57 (37)	42 (38)	2.2 (1.2–4.0)	2.0 (1.0–4.2)
Greater than 20 years ago	74 (28)	57 (37)	17 (15)	5.3 (2.6–11.0)	4.6 (2.0–10.5)
Missing	23 (9)	13 (8)	10 (9)	2.1 (0.8–5.4)	2.4 (0.8–6.8)

*aOR* adjusted Odds Ratio, *CI* Confidence Interval, *LSHDs* Local and/or State Health, *OR* Odds Ratio, *PRC* Prevention Research Center

Routes used for dissemination were indicated in response to the question: “What methods do you usually use to disseminate research findings?” Respondents were allowed to check all that applied. Rankings for the routes reported as having the largest impact on public health were obtained in response to the question: “Of the methods you use to disseminate the research findings, which one do you think generally has the most impact on public health practice or policy?” Respondents were allowed to choose one response from those selected in the previous question.

### Statistical analysis

Respondent characteristics and routes used for dissemination were examined with descriptive statistics. Frequencies for the routes reported as having the largest impact on public health were sorted from highest to lowest and provided a corresponding ranking (i.e. the largest frequency was given a ranking of 1 and so forth). Pearson χ^2^ or independent *t* tests were used to examine the association between dissemination and covariates. Logistic regression was used to calculate crude odds ratios (OR) and 95% confidence intervals (CI) for the association between respondent characteristics, facilitators, barriers, and the outcome of interest, dissemination to state and/or local health departments. In addition, multivariable logistic regression was used to calculate adjusted odds ratios (aOR) and 95% CIs. Estimates were adjusted for work place, graduate degree, and/or fellowship in public health, and year highest academic degree was received. Missing data was minimal (less than 10%) and incorporated into analyses. The literature suggests incorporating this data introduces little bias [27]. All analyses were conducted using SAS 9.3, SAS Institute Inc., Cary, NC.

**Table 2:** Logistic regression results for dissemination to LSHDs across facilitator characteristics and barriers to dissemination, 2012

	Disseminate Findings to United States LSHDs		
Predictors	(%)	Crude OR (95% CI)^a^	aOR (95% CI)^a,b^
Facilitator characteristics			
Individual-level			
Dissemination important to your own research	93	9.3 (4.5–19.2)	6.7 (2.9–15.3)
Formal training in health communication or access to someone with training	80	2.3 (1.3–4.0)	1.5 (0.8–2.8)
Worked in a practice or policy setting where research was applicable	77	3.3 (2.0–5.7)	2.8 (1.5–5.3)
Organizational-level			
Dissemination important to the work of your unit/department	75	3.6 (2.1–6.1)	2.7 (1.5–5.1)
Dissemination of findings to non-research audiences expected by funding agencies	63	2.8 (1.7–4.7)	2.6 (1.4–4.7)
Dissemination of findings to non-research audiences expected by employer	58	2.5 (1.5–4.1)	3.0 (1.6–5.9)
Dedicated person/team responsible for dissemination-related activities within unit/organization	57	1.5 (0.9–2.5)	1.7 (0.9–3.2)
Barriers			
Individual-level			
Uncertainty on how best to disseminate beyond professional conferences/publications	29	0.4 (0.3–0.7)	0.5 (0.3–0.9)
Lack of understanding about how to disseminate findings	25	0.7 (0.4–1.2)	0.8 (0.4–1.5)
Unsure which organizations want or would use the information	23	0.9 (0.5–1.6)	0.8 (0.4–1.6)
Uncertainty about the impact of dissemination	16	0.5 (0.3–0.9)	0.5 (0.2–1.1)
Uncertainty about what to disseminate	16	0.6 (0.3–1.0)	0.5 (0.2–1.1)
Hesitation/resistance to disseminate findings from a single study	16	0.3 (0.2–0.6)	0.5 (0.2–0.9)
Lack of information on audience make-up	10	1.1 (0.5–2.6)	1.2 (0.4–3.2)
Organizational-level			
Lack of financial resources for dissemination	63	2.0 (1.2–3.3)	2.2 (1.2–4.1)
Lack of staff time dedicated to dissemination	58	1.5 (0.9–2.4)	1.4 (0.8–2.4)
Lack of academic incentives for dissemination	36	0.5 (0.3–0.8)	0.6 (0.3–1.1)
Low priority for research dissemination in my unit/department	23	0.8 (0.4–1.3)	0.8 (0.4–1.6)
Dissemination activities not in study timelines	23	2.0 (1.0–3.8)	1.9 (0.9–4.2)
Lack of relationships with stakeholders	15	0.8 (0.4–1.6)	1.1 (0.5–2.3)

*aOR* adjusted Odds Ratio, *CI* Confidence Interval, *LSHDs* Local and/or State Health Departments, *OR* Odds Ratio

^a^ORs and aORs are for ‘Yes’ or ‘Very important/important’

^b^Model adjusted for work place, graduate degree and/or fellowship in public health, and year highest academic degree was received

## Results

### Respondent characteristics

Overall, the majority of respondents worked at a university, not affiliated with a PRC (41%), did not have a degree in public health (57%), and received their degree 10–20 years ago (37%; Table 1). Fifty-eight percent of respondents reported disseminating their research findings to LSHDs. After adjustments, there were a few characteristics associated with disseminating findings to LSHDs, notably, affiliation with a university PRC (aOR, 10.8; 95% CI, 3.3–35.9), the CDC (aOR, 9.5; 95% CI, 2.6–34.8), and respondents obtaining their degree more than 20 years ago (aOR, 4.6; 95% CI, 2.0–10.5).

### Facilitators to dissemination

In addition, individual- and organizational-level facilitators and barriers to dissemination were analyzed (Table 2). After adjusting for respondent characteristics, individual-level facilitators associated with disseminating to LSHDs included ever working in a practice or policy setting where research was applicable (aOR, 2.8; 95% CI, 1.5–5.3) and the importance of dissemination to the respondent’s own research (aOR, 6.7; 95% CI, 2.9–15.3). Organizational-level facilitators associated with disseminating to LSHDs included the expectation of dissemination by employers (aOR, 3.0; 95% CI, 1.6–5.9) or funding agencies (aOR, 2.6; 95% CI, 1.4–4.7), and the belief that dissemination was important to the work of the respondent’s unit/department (aOR, 2.7; 95% CI, 1.5–5.1).

### Barriers to dissemination

Individual-level barriers associated with disseminating to LSHDs included uncertainty on how best to disseminate beyond professional conferences or publications (aOR, 0.5; 95% CI, 0.3–0.9) and hesitation or resistance to disseminate findings from a single study (aOR, 0.5 95% CI, 0.2–0.9). For organizational-level barriers, lacking financial resources for dissemination (aOR, 2.2; 95% CI, 1.2–4.1) was also associated with dissemination to LSHDs.

### Routes for dissemination

The routes reported as having the largest impact on public health and the routes commonly used by respondents to disseminate their results were also explored (Table 3). Respondents who disseminated their findings to LSHDs ranked face-to-face meetings with stakeholders, academic journals, press releases, policy briefs, and media interviews as the top five routes they believed had the largest impact on public health. The dissemination routes reported as being most commonly used by the respondents were academic journals (100%), academic conferences (95%), reports issued to funders (78%), press releases (75%), seminars or workshops (71%), face-to-face meetings with stakeholders (68%), media interviews (60%), newsletters (59%), and other conferences (56%).

**Table 3:** Characteristics of routes used for dissemination as reported by public health researchers, 2012

	Disseminate findings to LSHDs		Disseminate to other target groups	
	Routes usually used to disseminate	Routes reported as having largest impact on public health	Routes usually used to disseminate	Routes reported as having largest impact on public health
	n = 154		n = 112	
Routes for dissemination	(%)	Ranking	(%)	Ranking
Academic journals	100	2	100	1
Academic conferences	95	9	89	6
Reports to funders	78	9	54	7
Press releases	75	3	45	5
Seminars or workshops	71	7	46	4
Face-to-face meetings with stakeholders	68	1	34	2
Media interviews	60	5	38	3
Newsletters	59	11	26	8
Other conferences	56	6	23	8
Policy briefs	39	3	8	8
Email alerts	32	11	8	11
Targeted mailings	24	11	5	11
Other	21	7	9	11
CD-ROMs	6	14	3	11

*LSHDs* Local and/or State Health Departments

## Discussion

Dissemination of evidence-based public health interventions to practitioners and other potential adopters is crucial to improving population health. Of the 266 researchers that responded to the survey, 58% reported disseminating their work to LSHDs. Affiliation with the CDC or a university PRC, and the respondent receiving their degree more than 20 years ago were important predictors of dissemination of research findings to LSHDs. After further inspection, 89% of university researchers affiliated with a PRC and 85% of CDC researchers disseminated their findings to LSHDs, compared to only 41% of university researchers not affiliated with a PRC and 32% of NIH researchers. The high percentage of CDC and university PRC researchers involved in dissemination to LSHDs may be attributed, in part, to the myriad evaluations of the program and resulting recommendations in which to work towards. A 1997 Institute of Medicine report recommended that PRCs focus on a community-based approach, concentrate more effort on dissemination and implementation of research findings, and specifically listed state and local health departments as a recipient of dissemination efforts [28, 29]. In 2008, the Association of Schools of Public Health (ASPH) published a set of recommendations from their assessment of the PRC program, to be used by the CDC and others [30]. The ASPH found that the CDC and PRCs showed a significant adaptation of the 1997 IOM recommendations and labelled the PRC program as the “*forefront of public health translational research*” [30]. Two of the recommendations to strengthen the program included enhancing collaboration with state and local health departments, and further dissemination of findings to the community, academia, and practice settings [30]. Dissemination is now considered a pivotal piece of the PRC program’s mission [28, 29, 31]. The findings from the present study suggest that the intersection of the IOM and ASPH recommendations, training programs, and support provided within the workplace helped drive the PRC researchers to succeed in their dissemination efforts.

The dissemination process includes both facilitators and barriers that exist on the individual- and organizational-level and serve to support or hinder the researcher during this course. The findings reveal that having ever worked in a practice or policy setting, importance of dissemination to the respondent’s unit or department, and expectations of dissemination to non-research audiences by employers or funding agencies were associated with dissemination to LSHDs. Funding agencies often state within grant award guidelines that researchers are expected to disseminate their research results, yet oftentimes either do not specify what dissemination should entail or only specify dissemination in the form of publication [14, 17, 18]. Specific guidelines stating dissemination expectations other than publication or “how-to” guides from funding agencies highlighting the process of disseminating public health findings to community partners or stakeholders may spur more researchers to disseminate beyond publication in academic journals. Performance metrics, which may include dissemination of research results, are also considered by some employers and may shed light on the significance of the current findings [14, 17, 18].

Respondents had lower odds of disseminating their results to LSHDs if they were uncertain on how best to disseminate beyond professional conferences or publications or if they faced hesitation or resistance to disseminate findings from a single research study. Gaps in the dissemination process could benefit from changes in infrastructure to incorporate ‘marketing and distribution systems’ or intermediaries to help increase communication, training, coordination, and to promote and deliver research discoveries and interventions to public health practitioners in LSHDs [16]. The concept of knowledge brokering (i.e. one-to-one technical assistance to facilitate dissemination) is a more active approach to dissemination and provides another strategy for removing some of the complexities of knowledge translation and assisting those who are uncertain on dissemination practices [32, 33]. However, hesitation or resistance to disseminate findings from a single study may not be unexpected, as Grimshaw et al. [34] suggested that single studies may be prone to more bias, and more evidence is usually required for changes to policy or practice than what is normally contained in a single study.

The finding that respondents had higher odds of disseminating to LSHDs if they lacked financial resources for dissemination was surprising. Of these respondents, 70% said dissemination was important to their unit or department, and 94% said dissemination was important to their own research, suggesting that personal commitment may play a role in overcoming this barrier.

Intriguing patterns emerged when respondents were asked to report their usual dissemination routes compared to routes they thought had the largest impact on public health. Academic journals, academic conferences, and reports to funders were the top three routes respondents usually used for dissemination. These results were consistent with the survey results reported by Wilson et al. [15] in the United Kingdom. Surprisingly, respondents who disseminated findings to LSHDs ranked face-to-face meetings with stakeholders as the route with the perceived largest impact on public health, yet only 68% of those respondents reported using this route. This discrepancy likely contributes to the gap between researchers and practitioners. Further, those who disseminated their findings to LSHDs ranked academic journals as the second route, and press releases and policy briefs tied for the third route reported as having the largest impact on public health. There appears to be a disconnect between the dissemination routes with the perceived largest public health impact and the reported routes used to distribute research results. Saul et al. [35] found that one of the main challenges for bridging research and practice is the “*need for greater understanding and communication between practitioners and researchers*,” and suggests that face-to-face meetings between researchers and practitioners would provide an opportunity for think tank sessions to occur in order to create mutual agendas and to increase collaboration between the two parties.

Respondents ranked academic journals as the route they believed had the second largest impact on public health, yet many public health departments may not have access to academic journals, specifically for those that require a subscription fee [36]. Fields et al. [37] found that, for local public health leaders, academic journals were the fourth most important method for learning about new research. Seminars or workshops, professional associations, and email alerts were the top three reported methods [37]. Researchers, on the other hand, often use academic journals to satisfy publication requirements for tenure promotions and to fulfil requirements by funders [14, 17, 18]. Academic journals are mainly targeted towards fellow researchers or those specifically interested in the topic at hand, which is why researchers may feel most comfortable disseminating research findings in journals [14]. Better understanding of researchers’ and practitioners’ needs along with increased communication between the two parties may aid in determining which dissemination routes would be the most effective for reaching LSHDs [33].

Researcher and practitioner ties need to continue to be strengthened in order to increase the number of public health researchers disseminating to LSHDs in the United States [38]. A study on the promotion of knowledge translation, conducted by Tetroe et al. [14], contained interviews with health research agency informants from various countries, including the United States. They discovered multiple examples of agencies that created networks or partnerships in order for researchers to have an avenue to share information and ideas. One such network, developed by the Agency for Healthcare Research and Quality, is a national program entitled “Put Prevention into Practice”, which serves to link practitioners to healthcare research to provide better and more effective services to patients [14, 39]. In addition, the Council on Linkages Between Academia and Public Health Practice (Council on Linkages) is an association of representatives from various public health organizations across the United States that is also functioning to strengthen researcher and practitioner ties [40]. One of their main initiatives is the development of an Academic Health Department Learning Community that serves to further link academia and practice by educating public health professionals on the practice of public health in state or local health departments [41]. The Academic Health Department Learning Community provides a forum for public health professionals to share various experiences, knowledge, and materials that they have obtained or developed through collaborations with state or local health departments [41]. Partnerships between academia and state health departments have been established in a number of states and localities [42]. These collaborations provide services such as community health assessments and continuing education for health department staff, while also providing field sites for university students [42, 43].

### Limitations

Some limitations should be noted in the study. The achieved response rate of 54.5% was slightly lower than the expected rate of 60%. Non-response bias might have been present and played a role in the lower response, but the obtained rate was comparable to a similar study in the United Kingdom, which achieved a 50% response rate [15]. There should also be some caution in interpreting these results due to wide confidence intervals for some of the variables, which could be due to a smaller sample size. In these instances, statistical significance may not suggest practical significance. These results are also based on self-reported data. Social desirability bias could have resulted in underreporting or overreporting of various characteristics and dissemination routes [44], though this may have been mitigated through the collection of anonymous responses. While it is possible that respondents’ perceptions of barriers and facilitators may differ from actual characteristics, it is arguable that these perceptions may drive respondents’ dissemination activities. In addition, other unmeasured organizational and contextual characteristics, such as culture and climate, may also be important predictors of researchers’ dissemination practices. Even though a definition for dissemination was provided in the survey, it cannot be ruled out that respondents interpreted the term other than what was intended. Finally, lead authors from publications were used as the study sample since they are commonly the corresponding authors, and email addresses were readily available for these contacts in PubMed. In cases where email addresses failed or the author was deceased, the second or last author was not contacted, and therefore, may have resulted in the findings being less generalizable to the entire research population.

### Future research

The current analysis combined state and local health departments since the focus of this article was dissemination of research findings to practitioners in public health departments overall. Future research could explore whether there are differences when analysing these as separate outcomes. The findings of the current study are intriguing and some warrant further research; namely, why researchers that lack financial resources for dissemination have higher odds for dissemination and the discrepancy between the routes used for dissemination and those with the perceived largest impact. Qualitative interviews with researchers would add richness to the quantitative findings noted.

## Conclusions

In summary, this study revealed that slightly over half of the respondents surveyed were disseminating their findings to LSHDs. This leaves considerable room to improve ties between the types of researchers in the present study and public health practice settings, and to decrease the barriers these researchers face during the dissemination process. When possible, researchers could utilize national programs, such as Put Prevention into Practice and the Academic Health Department Learning Community, as well as training programs and workshops to become more proficient in dissemination activities [39, 41, 45]. Finally, the role of academic institutions and funding agencies should not be underemphasized, as ultimately they provide both opportunities for training as well as the structure for incentives and awards that determine skills and resources necessary for prioritizing dissemination among the myriad of responsibilities of the typical academic researcher.

## References

[1] Wandersman A, Duffy J, Flaspohler P (2008). Bridging the gap between prevention research and practice: the interactive systems framework for dissemination and implementation. Am J Community Psychol.

[2] Clancy CM, Cronin K (2005). Evidence-based decision making: global evidence, local decisions. Health Aff (Millwood).

[3] Holmes B, Scarrow G, Schellenberg M (2012). Translating evidence into practice: the role of health research funders. Implement Sci..

[4] Fontanarosa PB, DeAngelis CD (2002). Basic science and translational research in JAMA. JAMA.

[5] Potter M, Quill B (2006). Demonstrating excellence in practice-based research for public health. Public Health Rep.

[6] Brownson RC, Jacobs JA, Tabak RG, Hoehner CM, Stamatakis KA (2013). Designing for dissemination among public health researchers: findings from a national survey in the United States. Am J Public Health.

[7] Harris JR, Cheadle A, Hannon PA (2012). A framework for disseminating evidence-based health promotion practices. Prev Chronic Dis..

[8] Eagle KA, Garson AJ, Beller GA, Sennett C (2003). Closing the gap between science and practice: the need for professional leadership. Health Aff (Millwood).

[9] Woolf SH (2008). The meaning of translational research and why it matters. JAMA.

[10] Woolf SH, Johnson RE (2005). The break-even point: when medical advances are less important than improving the fidelity with which they are delivered. Ann Fam Med.

[11] Fixsen DL, Naoom SF, Blase KA, Friedman RM, Wallace F (2005). Implementation research: A synthesis of the literature.

[12] McGlynn EA, Asch SM, Adams J (2003). The quality of health care delivered to adults in the United States. N Engl J Med.

[13] Lomas J (1993). Diffusion, dissemination, and implementation: who should do what?. Ann N Y Acad Sci..

[14] Tetroe JM, Graham ID, Foy R (2008). Health research funding agencies’ support and promotion of knowledge translation: an international study. Milbank Q.

[15] Wilson PM, Petticrew M, Calnan MW, Nazareth I (2010). Does dissemination extend beyond publication: a survey of a cross section of public funded research in the UK. Implement Sci..

[16] Kreuter MW, Bernhardt JM (2009). Reframing the dissemination challenge: a marketing and distribution perspective. Am J Public Health.

[17] The National Science Foundation. Proposal and Award Policies and Procedures Guide: Part II Award & Administration Guide. 2013:1–85. http://www.nsf.gov/pubs/policydocs/pappguide/nsf13001/aag_6.jsp#VID4. Accessed 6 Feb 2014.

[18] National Institutes of Health. Institutional Clinical and Translational Science Award (U54). 2012. http://grants.nih.gov/grants/guide/rfa-files/RFA-TR-12-006.html. Accessed 6 Feb 2014.

[19] Rosenstock L, Helsing K, Rimer BK (2011). Public health education in the United States: then and now. Public Health Rev.

[20] Gilliam A, Davis D, Barrington T, Lacson R, Uhl G, Phoenix U (2002). The value of engaging stakeholders in planning and implementing evaluations. AIDS Educ Prev.

[21] Roussos ST, Fawcett SB (2000). A review of collaborative partnerships as a strategy for improving community health. Annu Rev Public Health..

[22] Wright D, Anderson L, Brownson R (2008). Engaging partners to initiate evaluation efforts: Tactics used and lessons learned from the Prevention Research Centers program. Prev Chronic Dis.

[23] Brownson R, Colditz G, Proctor E (2012). Dissemination and Implementation Research in Health: Translating Science to Practice.

[24] Lomas J (1991). Words without action? The production, dissemination, and impact of consensus recommendations. Annu Rev Public Health.

[25] National Cancer Institute (2002). Designing for Dissemination: Conference Summary Report.

[26] Qualtrics: Survey Research Suite. 2012. http://www.qualtrics.com.

[27] Bennett DA (2001). How can I deal with missing data in my study?. Aust N Z J Public Health.

[28] Green LW (2007). The prevention research centers as models of practice-based evidence two decades on. Am J Prev Med.

[29] Stoto MA, Green LW, Bailey LA (1997). Linking research and public health practice: A review of CDC’s program of Centers for Research and Demonstration of Health Promotion and Disease Prevention.

[30] Association of Schools of Public Health (2008). Communities and academia working together: report of the Association of Schools of Public Health (ASPH) Prevention Research Centers (PRC) Blue Ribbon Panel.

[31] Franks AL, Brownson RC, Bryant C (2005). Prevention Research Centers: contributions to updating the public health workforce through training. Prev Chronic Dis.

[32] Dobbins M, Robeson P, Ciliska D (2009). A description of a knowledge broker role implemented as part of a randomized controlled trial evaluating three knowledge translation strategies. Implement Sci..

[33] Ward V, Hamer S, House A (2009). Knowledge brokering: the missing link in the evidence to action chain?. Evid Policy.

[34] Grimshaw JM, Eccles MP, Lavis JN, Hill SJ, Squires JE (2012). Knowledge translation of research findings. Implement Sci..

[35] Saul J, Duffy J, Noonan R (2008). Bridging science and practice in violence prevention: addressing ten key challenges. Am J Community Psychol.

[36] Harris JK, Allen P, Jacob RR, Elliott L, Brownson RC (2014). Information-seeking among chronic disease prevention staff in state health departments: use of academic journals. Prev Chronic Dis..

[37] Fields RP, Stamatakis KA, Duggan K, Brownson RC (2015). Importance of scientific resources among local public health practitioners. Am J Public Health.

[38] Mays GP, Hogg RA, Castellanos-Cruz DM, Hoover AG, Fowler LC (2013). Public health research implementation and translation: evidence from practice-based research networks. Am J Prev Med.

[39] Agency for Healthcare Research and Quality. Prevention Dissemination and Implementation. 2005. http://www.ahrq.gov/professionals/quality-patient-safety/quality-resources/tools/ppip/ppipabou.html. Accessed 7 Feb 2014.

[40] Public Health Foundation. Council on Linkages Between Academia and Public Health Practice. 2014. http://www.phf.org/programs/council/Pages/default.aspx/index.htm. Accessed 8 Feb 2014.

[41] Public Health Foundation. Academic Health Department Learning Community. 2014. http://www.phf.org/programs/AHDLC/Pages/Academic_Health_Department_Learning_Community.aspx. Accessed 8 Feb 2014.

[42] Livingood WC, Goldhagen J, Little WL, Gornto J, Hou T (2007). Assessing the status of partnerships between academic institutions and public health agencies. Am J Public Health.

[43] Erwin PC, Barlow P, Brownson RC, Amos K, Keck CW (2016). Characteristics of academic health departments: initial findings from a cross-sectional survey. J Public Health Manag Pract.

[44] Fisher RJ (1993). Social desirability bias and the validity of indirect questioning. J Consum Res.

[45] Meissner HI, Glasgow RE, Vinson CA (2013). The U.S. training institute for dissemination and implementation research in health. Implement Sci.

